# CGRP induces migraine-like symptoms in mice during both the active and inactive phases

**DOI:** 10.1186/s10194-021-01277-9

**Published:** 2021-06-30

**Authors:** Anne-Sophie Wattiez, Olivia J. Gaul, Adisa Kuburas, Erik Zorrilla, Jayme S. Waite, Bianca N. Mason, William C. Castonguay, Mengya Wang, Bennett R. Robertson, Andrew F. Russo

**Affiliations:** 1 Department of Molecular Physiology and Biophysics, University of Iowa, 51 Newton Rd, Iowa City, IA 52242 USA; 2 Center for the Prevention and Treatment of Visual Loss, Veterans Administration Health Center, Iowa City, IA 52246 USA; 3 Neuroscience Program, University of Iowa, Iowa City, IA 52242 USA; 4 Present address: Brain and Behavior Sciences, Center for Advanced Pain Studies, University of Texas at Dallas, 800 West Campbell Rd, Richardson, TX 75080 USA; 5 Department of Pharmacology, University of Iowa, Iowa City, IA 52242 USA; 6 Department of Neurology, University of Iowa, Iowa City, IA 52242 USA

**Keywords:** Migraine, Circadian patterns, CGRP, Light aversion, Movement, Wheel running

## Abstract

**Background:**

Circadian patterns of migraine attacks have been reported by patients but remain understudied. In animal models, circadian phases are generally not taken into consideration. In particular, rodents are nocturnal animals, yet they are most often tested during their inactive phase during the day. This study aims to test the validity of CGRP-induced behavioral changes in mice by comparing responses during the active and inactive phases.

**Methods:**

Male and female mice of the outbred CD1 strain were administered vehicle (PBS) or CGRP (0.1 mg/kg, i.p.) to induce migraine-like symptoms. Animals were tested for activity (homecage movement and voluntary wheel running), light aversive behavior, and spontaneous pain at different times of the day and night.

**Results:**

Peripheral administration of CGRP decreased the activity of mice during the first hour after administration, induced light aversive behavior, and spontaneous pain during that same period of time. Both phenotypes were observed no matter what time of the day or night they were assessed.

**Conclusions:**

A decrease in wheel activity is an additional clinically relevant phenotype observed in this model, which is reminiscent of the reduction in normal physical activity observed in migraine patients. The ability of peripheral CGRP to induce migraine-like symptoms in mice is independent of the phase of the circadian cycle. Therefore, preclinical assessment of migraine-like phenotypes can likely be done during the more convenient inactive phase of mice.

**Supplementary Information:**

The online version contains supplementary material available at 10.1186/s10194-021-01277-9.

## Background

Clinicians and patients have anecdotally reported temporal patterns (seasonal, circadian) of migraine attacks, leading to terminology such as cyclical, nocturnal, or weekend migraines [1, 2]. While little experimental data exists, most studies report a peak of onset of migraine attacks either in the early morning [3, 4] or late at night [5]. Fewer reports describe biphasic patterns [6], or a peak around the middle of the day [1]. Those contradictory results have been reviewed by Bkasa et al., [7] who highlight the methodological differences between the studies that may contribute to the differences (number of patients, prospective or retrospective nature, specific populations, type of headache, use of medication). Recently, a study showed that migraine patients have a higher likelihood to be “morning larks” (i.e. go to sleep and wake early) than a non-migraine subjects, and in turn, that “morning larks” were more likely to have migraines in the morning while “night owls” (i.e. go to sleep and wake late) were more likely to have migraines in the evening [6]. Overall, circadian patterns exist in migraine pathophysiology and need to be further studied in patients.

The association between migraine and sleep disorders is well known [8, 9], and is likely bidirectional [10, 11]. In fact, the onset of migraine attacks in the early morning has already been linked to insomnia [12]. Of importance, sleep can be both a treatment [13] and a trigger for migraine [14, 15]; however, sleep disruptions may contribute to the chronobiology of migraine or alternatively sleep disruptions could result from migraine. This conundrum highlights the need for studies on sleep patterns, circadian patterns, and chronobiology in migraine.

Preclinical migraine studies are mainly performed in rodents, which are nocturnal animals. While there are preclinical studies that specifically look at the relation between migraine and sleep in transgenic models [16, 17], circadian patterns and sleep are not often taken into consideration with rodent assays. Because it is common practice due to convenience, most preclinical studies are performed during the daytime, which corresponds to the rodent inactive phase. It is therefore legitimate to wonder if results obtained in rodents during inactive phase would be similar if the experiments were performed during the active phase, either at night or with animals housed on an inverted cycle. This concern is magnified by the fact that motility is a variable for many behavioral assays [18]. Furthermore, testing during the day time causes acute sleep disruption, which is known to increase pain sensitivity [11]. Finally, the question is particularly relevant when conducting light aversive behavior assays because this test contains a light component that may add to the complexity of chronobiology and sleep. It is known that the peripheral administration of calcitonin gene-related peptide (CGRP) induces light aversion in mice when the assay is performed during the day (inactive phase) [19]. In the present study, we aimed to compare the effects of CGRP when administered during the active and inactive phases of the circadian cycle in an outbred strain of mice.

## Methods

### Animals

Male and female CD1 (Charles River, USA) mice were used. Mice were 8–9 weeks of age upon arrival at our facility and allowed to acclimate for a week before use. Since similar results were observed in male and female mice, data from both sexes were combined for all studies; however, we were not powered to detect subtle sex differences. In all scatter plots figures, empty symbols represent females, and full symbols represent males. Mice were housed in groups of 4 per cage, on a 12 h light cycle with food and water ad libitum. Lights were turned on at 6 AM and turned off at 6 PM. The goal of the present study was to assess the response of animals to migraine triggers a few hours after the onset of their active or inactive phase, therefore most experiments were run 2 to 4 h after the change in the light cycle. For all experiments, investigators were blinded to drug treatment and animals randomized (block randomization) to each treatment group prior to commencement of experiments. For each assay, mice were brought to the experimental room 1 h prior to the beginning of the experiment for acclimation. Animal procedures were approved by the University of Iowa Animal Care and Use Committee and performed in accordance with the standards set by the National Institutes of Health and the ARRIVE guidelines.

### Drug administration

All drugs were administered by intraperitoneal (i.p.) injection at 10 μl/g bodyweight with a 30 g × 0.5 needle. Rat α-CGRP (Sigma-Aldrich, USA) was administered at 0.1 mg/kg as reported in our previous studies [19]. CGRP was diluted in modified Dulbecco PBS (Hyclone, GE Healthcare Life Science, USA), which was also used for vehicle groups. Except for the wheel assays for which animals were placed in the apparatus immediately after injection, for all other behavioral experiments, mice were allowed to recover for 30 min in their home cages before testing, in accordance with previously published data [19, 20].

### Light aversion and motility assays

The light/dark and resting data were collected using Activity Monitor version 7.06 (Med Associate Inc) from twelve chambers as previously described [19, 21]. Mice were pre-exposed to the chamber once as a baseline measurement, then tested with bright light (25,000 lx) [19, 21]. Assays started at 10 AM or 8 PM as indicated on the graphs. Data were collected for 30 min and analyzed in sequential 5 min intervals. This assay depends on the exploratory drive of the animals and is limited to 30 min since mice tend to stop exploring if left in the chamber for longer times. The time in light was reported as the mean +/− SEM of all the mice at each interval and as the mean +/− SEM of the average time per interval for each individual mouse.

Resting data were collected during the light aversion assay and were calculated as the percentage of time spent not moving (not breaking any new infrared beams). Resting data in the light and dark zones were normalized to time spent in each zone and expressed as % of time.

### Homecage activity monitoring

This assay was performed to assess the activity of animals over 23 h, with beginning of the test at different times of the active or inactive phase. The animal’s activity was recorded using Laboratory Animal Behaviour Observation, Registration and Analysis System (LABORAS™, Metris B.V., Hoofddorp, Netherlands), a non-invasive activity monitoring system consisting of an automated platform that detects vibration and force to determine normal rodent behaviors [22]. Thirty min after the injection of either CGRP (0.1 mg/kg, i.p.) or PBS, mice were placed in individual cages similar to their homecage with unlimited access to food and water. Assays started at 10 AM, 2 PM or 8 PM depending on the experiment. The same animals were used for the 3 repetitions of this assay. The activity data from the platforms were computed for 23 h. The activity was reported as the mean +/− SEM of the distance (m) traveled by mice per hour. Additionally, the distance travelled by mice during the first 30 min of the assay was reported (right panel of each graph).

### Wheel activity assay

Mice were pre-exposed to the wheels once prior to testing in order to habituate them to the new environment and learn how to use the wheel. On testing day, mice were individually enclosed in wheels for 2 h*.* We have previously shown that CGRP induces migraine-like phenotypes for 60 to 75 min, therefore a duration of 2 h is enough to show the totality of the effect of CGRP. Mice were free to stay immobile, walk, or run in the wheel. Assays were started at 8 AM or 10 PM. After 2 h, mice were placed back in their home cage. Number of wheel revolutions per 5 min were collected using the Activity Wheel Data Collection Utility software coupled to the wheels (Med Associate Inc). Wheel revolutions were reported as mean +/− SEM of all the mice at each interval.

### Automated measurement of squinting behavior

The squinting behavior was measured using video imaging and mouse facial detection as described [23]. Briefly, mice were acclimated to a customized gentle collar restraint prior to experimentation [24]. Following a 5 min baseline video recording, CGRP (0.1 mg/kg i.p.) or vehicle (PBS) were administered, and mice were returned to their home cage. Mice were restrained once more 30 min post-injection and recorded for squint assessment over 5 min. Pixel area measurement for the right eye palpebral fissure was derived every 0.1 s (10 frames per sec) in the recordings using a trained facial detection software with the resulting values compiled with custom MATLAB script. Half of the mice were tested during the day, and then during the night a week later. The other half was tested during the night first, and during the day a week later.

### Statistics

Data were analyzed using GraphPad Prism 8.4 software (RRID: SCR_002798). When data are plotted as a function of time (line graphs), a two-way repeated measure ANOVA was performed (factors time and treatment) including all the time-points presented in the figures. When needed, a Sidak’s multiple-comparison test was performed to compare the effect of each treatment at each time point, and symbols on the figure indicate the difference of each treatment group compared to the control group, at each time-point. When data are plotted as averages for each treatment (scatter plot graphs), an unpaired t-test was performed to compare the effect of CGRP to vehicle. All statistics are reported in Table 1.

**Table 1:** Statistical analysis

Figure #	Analysis		Statistics
Figure 1B Left panel	Two-way RM ANOVA	Interaction factor	*F*_(22,1276)_ = 0.759, *p =* 0.779
		Time factor	*F*_(11.8684.7)_ = 34.38, *p* < 0.0001
		Treatment factor	*F*_(1,58)_ = 1.718, *p* = 0.195
Figure 1B Right panel	Unpaired t-test		*p* = 0.0063
Figure 1C Left panel	Two-way RM ANOVA	Interaction factor	*F*_(22,1276)_ = 1.191, *p =* 0.245
		Time factor	*F*_(10.2, 591.7)_ = 35.78, *p* < 0.0001
		Treatment factor	*F*_(1,58)_ = 1.366, *p* = 0.247
Figure 1C Right panel	Unpaired t-test		*p* = 0.0035
Figure 1D Left panel	Two-way RM ANOVA	Interaction factor	*F*_(22,1276)_ = 1.317, *p =* 0.148
		Time factor	*F*_(9.22,531.1)_ = 54.93, *p* < 0.0001
		Treatment factor	*F*_(1,58)_ = 0.196, *p* = 0.660
Figure 1D Right panel	Unpaired t-test		*p* = 0.0042
Figure 2A 10 am (up^a^)	Two-way RM ANOVA	Interaction factor	*F*_(23,299)_ = 1.012, *p =* 0.449
		Time factor	*F*_(1.04,13.58)_ = 73.07, *p* < 0.0001
		Treatment factor	*F*_[_1_,_ 13_]_=1.063, *p* = 0.321
Figure 2A 8 pm (up^a^)	Two-way RM ANOVA	Interaction factor	*F*_(24,264)_ = 0.769, *p =* 0.779
		Time factor	*F*_(1.07,11.77)_ = 117.7, *p* < 0.0001
		Treatment factor	*F*_[_1_,_ 11_]_=0.757, *p* = 0.403
Figure 2B 8 pm (up^a^)	Two-way RM ANOVA	Interaction factor	*F*_(48,600)_ = 3.025, *p* < 0.0001
		Time factor	*F*_(1.57,19.69)_ = 68.33, *p* < 0.0001
		Treatment factor	*F*_[_1_,_ 13_]_=7.246, *p* = 0.0185
Figure 2B 10 am (up)	Two-way RM ANOVA	Interaction factor	*F*_(48,574)_ = 8727, *p* = 0.715
		Time factor	*F*_(1.36,16.27)_ = 88.28, *p* < 0.0001
		Treatment factor	*F*_[_1_,_ 13_]_=1.335, *p* = 0.2687
Figure 2C	Unpaired t-test first hour		*p* = 0.0242
	Unpaired t-test second hour		*p* = 0.5009
Figure 2D	Unpaired t-test first hour		*p* = 0.0213
	Unpaired t-test second hour		*p* = 0.1474
Figure 3B Left panel	Baseline 10 AM Two-way RM ANOVA		
		Interaction factor	*F*_(5,90)_ = 2.813, *p =* 0.0209
		Time factor	*F*_(3.435,61.83)_ = 2.371, *p* = 0.071
		Treatment factor	*F*_[_1_,_ 18_]_=0.3919, *p* = 0.539
	Test 10 AM Two-way RM ANOVA		
		Interaction factor	*F*_(5,90)_ = 2.813, *p =* 0.0006
		Time factor	*F*_(3.048,54.86)_ = 2.228, *p* = 0.094
		Treatment factor	*F*_[_1_,_ 18_]_=14, *p* = 0.0015
	Test 8 PM Two-way RM ANOVA		
		Interaction factor	*F*_(5,90)_ = 1.048, *p =* 0.395
		Time factor	*F*_(3.492,62.85)_ = 8.281, *p* < 0.0001
		Treatment factor	*F*_[_1_,_ 18_]_=18.57, *p* = 0.0004
Figure 3B Right panel	Unpaired t-tests	Baseline 10 am	*p* = 0.539
		Test 10 am	*p* = 0.0015
		Test 8 pm	*p* = 0.0004
Figure 4B Left panel	Baseline 8 PM Two-way RM ANOVA		
		Interaction factor	*F*_(5,90)_ = 1.726, *p =* 0.137
		Time factor	*F*_(4.01,72,18)_ = 3579, *p* = 0.0101
		Treatment factor	*F*_[_1_,_ 18_]_=0.4585, *p* = 0.507
	Test 8 PM Two-way RM ANOVA		
		Interaction factor	*F*_(5,90)_ = 1.545, *p =* 0.184
		Time factor	*F*_(3.882,69.88)_ = 5.956, *p* = 0.0004
		Treatment factor	*F*_[_1_,_ 18_]_=19, *p* = 0.0004
	Test 10 AM Two-way RM ANOVA		
		Interaction factor	*F*_(5,90)_ = 0.5679, *p =* 0.724
		Time factor	*F*_(2.612,27.02)_ = 0.7879, *p* = 0.491
		Treatment factor	*F*_[_1_,_ 18_]_=6.589, *p* = 0.0194
Figure 4B Right panel	Unpaired t-tests	Baseline 8 pm	*p* = 0.507
		Test 8 pm	*p* = 0.0004
		Test 10 am	*p* = 0.0004
Figure 5A	Baseline 10 AM Two-way RM ANOVA		
		Interaction factor	*F*_(5,90)_ = 1.177, *p =* 0.327
		Time factor	*F*_(3.586, 64.55)_ = 25.17, *p* < 0.0001
		Treatment factor	*F*_[_1_,_ 18_]_=0.006, *p* = 0.936
	Test 10 AM Two-way RM ANOVA		
		Interaction factor	*F*_(5,90)_ = 3.653, *p =* 0.0047
		Time factor	*F*_(3.714,66.84)_ = 12.94, *p* < 0.0001
		Treatment factor	*F*_[_1_,_ 18_]_=27.78, *p* < 0.0001
	Test 8 PM Two-way RM ANOVA		
		Interaction factor	*F*_(5,90)_ = 0.4425, *p =* 0.818
		Time factor	*F*_(2.98,53.64)_ = 41.79, *p* < 0.0001
		Treatment factor	*F*_[_1_,_ 18_]_=26.79, *p* < 0.0001
Figure 5B	Baseline 10 AM Mixed-effects analysis		
		Interaction factor	*F*_(5,87)_ = 0.4525, *p =* 0.810
		Time factor	*F*_(2.659,46.26)_ = 5.407, *p* = 0.0039
		Treatment factor	*F*_[_1_,_ 18_]_=0.025, *p* = 0.876
	Test 10 AM Mixed-effects analysis		
		Interaction factor	*F*_(5,73)_ = 1.831, *p =* 0.117
		Time factor	*F*_(3.419,49.92)_ = 7.620, *p* = 0.0002
		Treatment factor	*F*_[_1_,_ 18_]_=1.271, *p* = 0.274
	Test 8 PM Mixed-effects analysis		
		Interaction factor	*F*_(5,61)_ = 4.364, *p =* 0.0018
		Time factor	*F*_(1.896,24.22)_ = 8.867, *p* = 0.0013
		Treatment factor	*F*_[_1_,_ 18_]_=0.0172, *p* = 0.897
Figure 5C	Baseline 8 PM Two-way RM ANOVA		
		Interaction factor	*F*_(5,90)_ = 0.9072, *p =* 0.907
		Time factor	*F*_(3.335,60.04)_ = 29.30, *p* < 0.0001
		Treatment factor	*F*_[_1_,_ 18_]_=0.0558, *p* = 0.8159
	Test 8 PM Two-way RM ANOVA		
		Interaction factor	*F*_(5,90)_ = 0.86, *p =* 0.511
		Time factor	*F*_(3.635,65.44)_ = 32.49, *p* < 0.0001
		Treatment factor	*F*_[_1_,_ 18_]_=7.239, *p* = 0.0150
	Test 10 AM Two-way RM ANOVA		
		Interaction factor	*F*_(5,90)_ = 1.349, *p =* 0.2510
		Time factor	*F*_(3.005,54.08)_ = 18.67, *p* < 0.0001
		Treatment factor	*F*_[_1_,_ 18_]_=3.494, *p* = 0.0779
Figure 5D	Baseline 8 PM Mixed-effects analysis		
		Interaction factor	*F*_(5,86)_ = 1.114, *p =* 0.359
		Time factor	*F*_(2.837,48.79)_ = 12.33, *p* < 0.0001
		Treatment factor	*F*_[_1_,_ 18_]_=0.2415, *p* = 0.629
	Test 8 PM Mixed-effects analysis		
		Interaction factor	*F*_(5,77)_ = 1.786, *p =* 0.126
		Time factor	*F*_(3.619,55.74)_ = 7.105, *p* = 0.0002
		Treatment factor	*F*_[_1_,_ 18_]_=0.1405, *p* = 0.712
	Test 10 AM Mixed-effects analysis		
		Interaction factor	*F*_(5,82)_ = 1.142, *p =* 0.3449
		Time factor	*F*_(32.051,33.63)_ = 5.67, *p* = 0.0071
		Treatment factor	*F*_[_1_,_ 18_]_=0.1632, *p* = 0.6910
Figure 6B	Two-way RM ANOVA		
		Interaction factor	*F*_[_1_,_ 25_]_=20.95, *p* < 0.0001
		Time factor	*F*_[_1_,_ 25_]_=4.953, *p* = 0.0034
		Treatment factor	*F*_[_1_,_ 25_]_=7.707, *p* = 0.0095
Figure 6D	Two-way RM ANOVA		
		Interaction factor	*F*_[_1_,_ 26_]_=13.66, *p* = 0.0009
		Time factor	*F*_[_1_,_ 26_]_=7.585, *p* = 0.0099
		Treatment factor	*F*_[_1_,_ 26_]_=9.298, *p* = 0.0008

*RM* Repeated measures; up^a^: underpowered separate when plotted alone; multiple comparison analysis detailed in figure legends when needed

## Results

### CGRP administration only affects activity during the first hour after administration independently of the time of the day

**Fig. 1:**
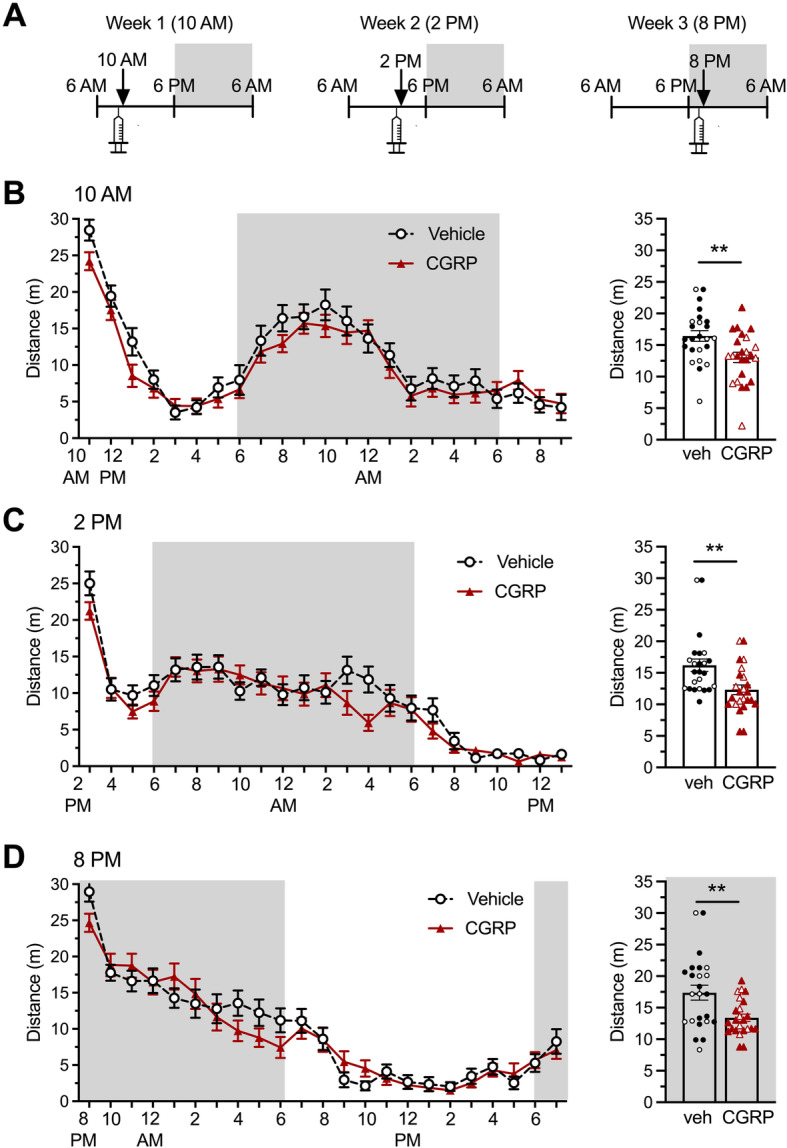
Peripheral CGRP administration does not change the overall activity of mice throughout the circadian cycle but decreases activity during the first hour after administration. Distance traveled by CD1 mice was measured during 23 consecutive hours starting 30 min after injection of vehicle (PBS, i.p., *n* = 22) or CGRP (0.1 mg/kg i.p., *n* = 24), using LABORAS™. Each experiment was done a week apart over 3 consecutive weeks with the same animals. In all panels, grey areas represent the active phase of the animals when lights of the facility were turned off (night). Empty symbols represent females, and full symbols represent males. There were no statistical differences between males and females. **A** Schematics of the experimental protocol. **B** On week 1, CGRP was administered at 9:30 am and assay started at 10:00 am. Left panel shows the time course of distance travelled over 23 h. Two-way (factors: time x treatment) ANOVA (ns). The right panel shows the average of the distance traveled during the first 30 min of the week 1 test. Unpaired t-test **p = 0.0063 comparing CGRP with vehicle group. **D** On week 2, CGRP was administered at 1:30 pm and assay started at 2:00 pm. Left panel shows the time course of distance travelled over 23 h. Two-way (factors: time x treatment) ANOVA (ns). The right panel shows the average of the distance traveled during the first 30 min of the week 2 test. Unpaired t-test **p = 0.0035 comparing CGRP with vehicle group. **F** On week 3, CGRP was administered at 7:30 pm and assay started at 8:00 pm. Left panel shows the time course of distance travelled over 23 h. Two-way (factors: time x treatment) ANOVA (interaction and treatment factors ns). The right panel shows the average of the distance traveled during the first 30 min of the week 3 test. Unpaired t-test **p = 0.0042 comparing CGRP with vehicle group

The effect of peripheral administration of CGRP on activity was assessed using the LABORAS™ system over 23 h at three different times of administration. On week 1, mice were injected with CGRP (0.1 mg/kg, i.p.) or vehicle (PBS i.p.) at 9:30 AM and placed individually in the activity cages at 10 AM for monitoring (Fig. 1A). On week 2, this protocol was repeated using the same mice, given the same treatments, but injected at 1:30 PM and monitored starting at 2 PM. Finally, on week 3, the mice were injected at 7:30 PM and monitored starting at 8 PM. The distance traveled by mice every hour during the 23 h-monitoring period of each treatment time was measured (Fig. 1B, C, D left panels). The first 30 min of distance traveled is shown for individual mice (Fig. 1B, C, D right panels). When data is analyzed over the 23 h, the overall treatment factor (CGRP vs. vehicle-injected groups) remained non-significant (see Table 1 for detailed statistics). However, CGRP decreased the distance travelled at certain time-points and did so consistently during the first hour after administration. For this reason, and because it corresponds to the time at which light aversion is assessed in subsequent experiments, the first 30 min of monitoring is presented as scatter plot bar graphs. During the first 30 min of the assay, CGRP significantly decreased the distanced traveled by mice independently of the time of the circadian cycle at which they were injected, and therefore, independently of their active and inactive phase. Of note, there were no sex differences observed for this assay.

Additionally, these results also show a burst of activity always present at the beginning of the assay, no matter what time of the circadian cycle the monitoring starts.

**Fig. 2:**
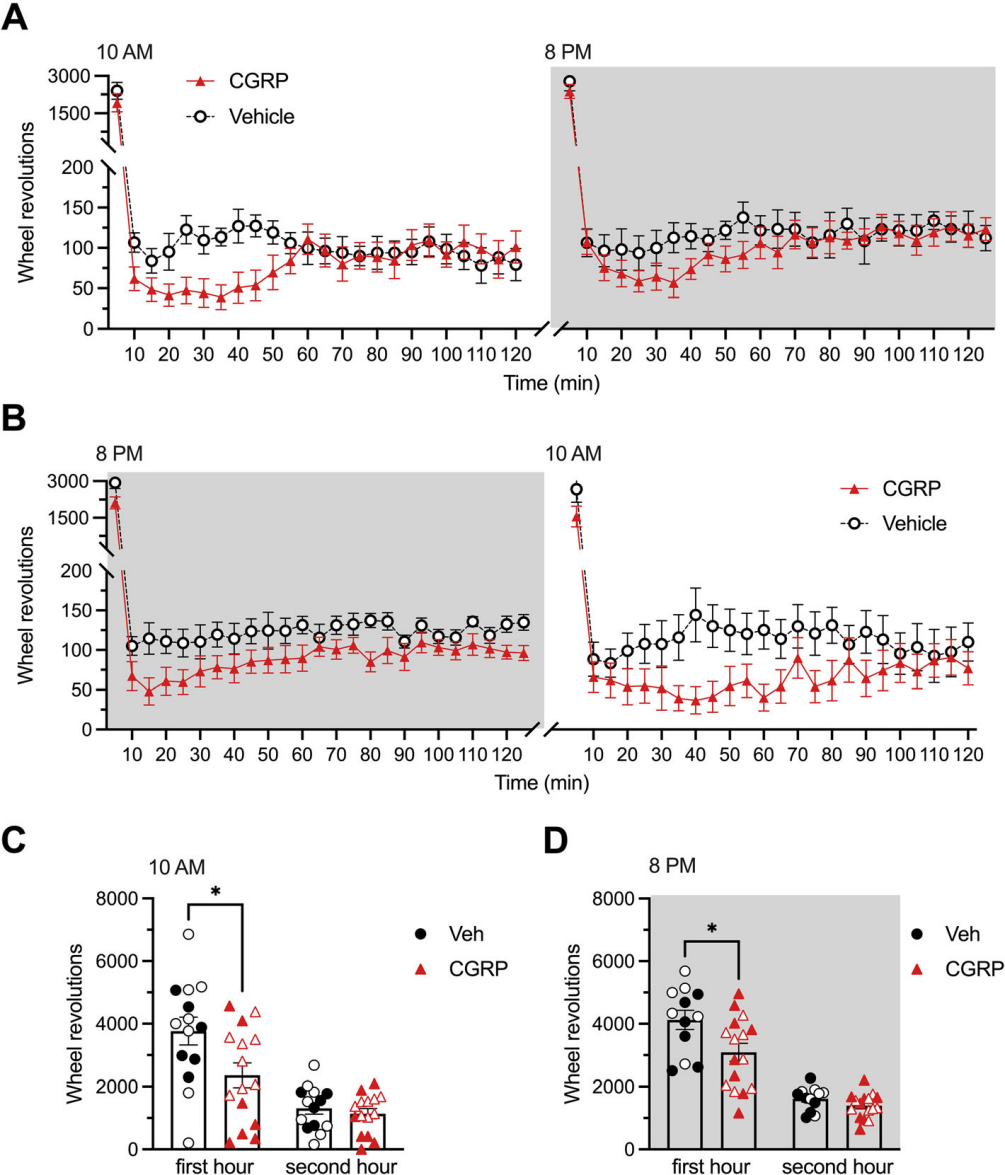
Peripheral CGRP administration depresses wheel activity during both the inactive and active phases. In all panels, grey areas represent the active phase of the animals when lights of the facility were turned off (night). Empty symbols represent females, and full symbols represent males. There were no statistical differences between males and females. Number of wheel revolutions by CD1 mice was measured for 2 h starting immediately after treatment administration. **A** The first cohort of mice was tested at 10 AM first, then at 8 PM a week later. Animals were administered with vehicle (PBS, i.p., *n* = 7) or CGRP (0.1 mg/kg i.p., *n* = 8) before being placed in the wheels. **B** The second cohort of mice was tested at 8 PM first, then at 10 AM a week later. Animals were administered with vehicle (PBS, i.p., n = 7) or CGRP (0.1 mg/kg i.p., n = 8) before being placed in the wheels. **C** Data accumulated at 10 AM from the two cohorts in A and B was pooled together and analyzed per hour. **D** Data accumulated at 8 PM from the two cohorts in A and B was pooled together and analyzed per hour. Two-way (factors: time x treatment) ANOVA (interaction and treatment factors ns) for panels A and B (underpowered). Unpaired t-test (*p < 0.05) for panels C and D

While LABORAS™ assesses basic activity, more specific innate activity can also be measured using a wheel. In a second experiment, two different cohorts of mice were tested in parallel. The first cohort was placed in an enclosed wheel immediately after the administration of CGRP (0.1 mg/kg, i.p.) or vehicle (PBS i.p.) at 10 AM. Four days later, the same mice were given the same treatments at 8 PM and then immediately placed in the wheels. Wheel testing lasted 2 h, and data were recorded every 5 min (Fig. 2A). Similarly to the basic activity data, the mice had a high number of wheel revolutions during the first few minutes, and then the number of revolutions decreased for the remainder of the assay. CGRP administration decreased the number of wheel revolutions compared to the vehicle group during the first hour of the experiment, both at 10 AM and at 8 PM. The second cohort of mice was tested in reverse, with first an injection at 8 PM, and 4 days later at 10 AM. The results (Fig. 2B) are identical to the first cohort. Since those cohorts are not powered as individual experiments, the results were then pooled by time of administration of treatments. CGRP induced a significant decrease in wheel revolution compared to vehicle during the first hour but not the second hour following injection at either 10 AM (Fig. 2C) or 8 PM (Fig. 2D). Overall, CGRP significantly decreased the number of wheel revolutions during the first hour after injection independently of the active or inactive phase of the mice. Once again, there were no sex differences in this assay.

### CGRP administration induces light aversion independently of the time of the day

**Fig. 3:**
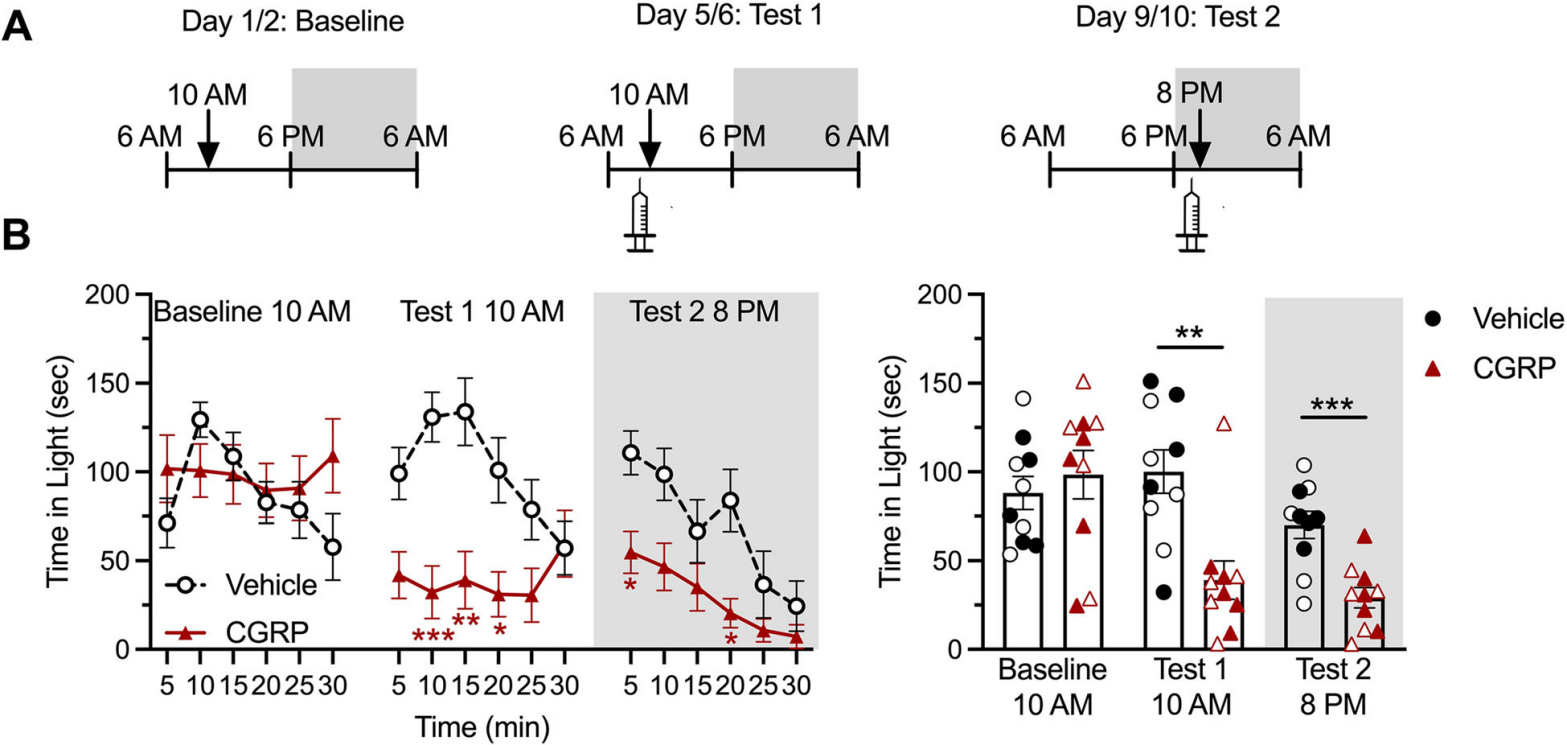
Peripheral CGRP induces light aversion during both the inactive and active phases. In all panels, grey areas represent the active phase of the animals when lights of the facility were turned off (night). Empty symbols represent females, and full symbols represent males. There were no statistical differences between males and females. **A** Experimental design: mice were first baselined during their inactive phase (experiment starting at 10:00 am), and then exposed to treatments on the first test day during their inactive phase (10 am), and second test day during their active phase (experiment starting at 8:00 pm). **B** Left panel. Time spent in the light zone by CD1 mice during sequential exposures to the light/dark chamber at 25,000 lx. On test days, animals were tested 30 min after injection of vehicle (PBS, i.p., *n* = 10) or CGRP (0.1 mg/kg i.p., *n* = 10). Two-way (factors: time x treatment) ANOVA Sidak’s multiple comparison test *p < 0.05, **p < 0.01 and ***p < 0.001 compared to corresponding time point in vehicle group. Right panel. Mean time (± SEM) spent in the light zone per 5 min interval for individual mice during the same experiment represented in (A). Unpaired t-test **p < 0.01 and ***p < 0.001 comparing CGRP group with corresponding vehicle group. **C** Mice were first baselined during their active phase (experiment starting at 8:00 pm), and then exposed to treatments on the first test day during their active phase (8 pm), and second test day during their inactive phase (experiment starting at 10:00 am). Two-way (factors: time x treatment) ANOVA, Sidak’s multiple comparison test *p < 0.05, **p < 0.01 and ***p < 0.001 compared to corresponding time point in vehicle group. **D** Mean time (± SEM) spent in the light zone per 5 min interval for individual mice during the same experiment represented in (C). Unpaired t-test **p < 0.01 and ***p < 0.001 comparing CGRP group with corresponding vehicle group.

**Fig. 4:**
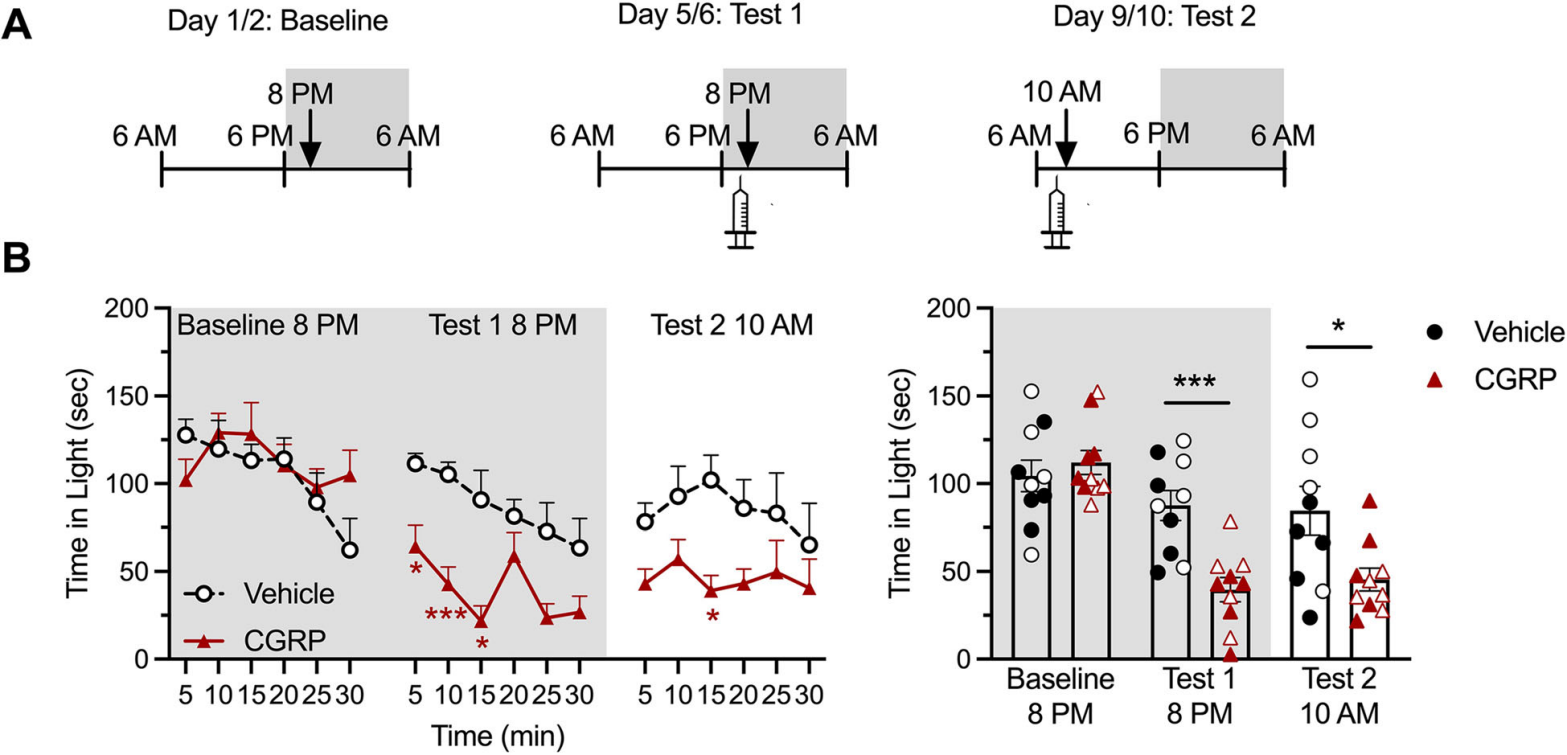
Peripheral CGRP induces light aversion during both the active and inactive phases (reverse testing from Fig. 3). In all panels, grey areas represent the active phase of the animals when lights of the facility were turned off (night). Empty symbols represent females, and full symbols represent males. There were no statistical differences between males and females. **A** Experimental design: Mice were first baselined during their active phase (experiment starting at 8:00 pm), and then exposed to treatments on the first test day during their active phase (8 pm), and second test day during their inactive phase (experiment starting at 10:00 am). **B** Left panel. Time spent in the light zone by CD1 mice during sequential exposures to the light/dark chamber at 25,000 lx. On test days, animals were tested 30 min after injection of vehicle (PBS, i.p., n = 10) or CGRP (0.1 mg/kg i.p., n = 10). Two-way (factors: time x treatment) ANOVA, Sidak’s multiple comparison test *p < 0.05, **p < 0.01 and ***p < 0.001 compared to corresponding time point in vehicle group. Right panel. Mean time (± SEM) spent in the light zone per 5 min interval for individual mice during the same experiment represented in (C). Unpaired t-test **p < 0.01 and ***p < 0.001 comparing CGRP group with corresponding vehicle group

Two cohorts of mice were run in parallel in order to investigate the effect of the circadian cycle on CGRP-induced light aversion. Each cohort underwent 3 light aversion trials: a baseline, and two tests comparing CGRP versus vehicle, each separated by 2 to 3 days (Figs. 3 and 4). One cohort was run during the light phase of the circadian cycle for baseline and test 1, and during the dark phase of the cycle for test 2 (Fig. 3A). The second cohort was reversed: baseline in the dark phase, 1st test in the dark phase, and 2nd test in the light phase (Fig. 4A). The first cohort shows that CGRP induces a decrease in time spent in the light both when animals were tested during the day, and then again during the night (Fig. 3B left panel for time course effect, and right panel for average over 30 min). The second cohort showed similar decrease in time spent in the light after CGRP administration first during the night, and then during the day (Fig. 4B). Of note, there were no sex differences in this assay.

**Fig. 5:**
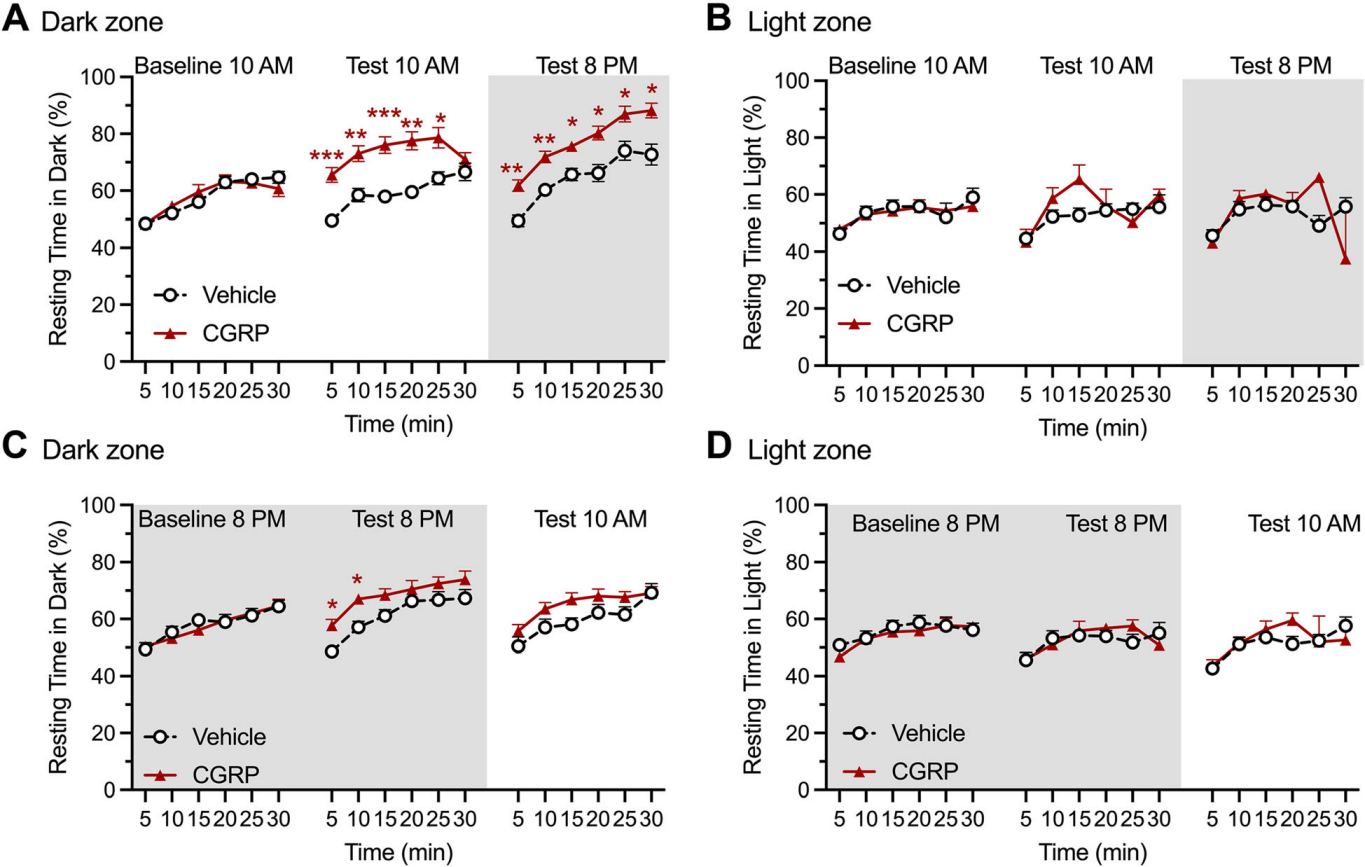
Peripheral CGRP increases the time resting in the dark during both the active and inactive phases. In all panels, grey areas represent the active phase of the animals when lights of the facility were turned off (night). Percent time resting in the dark and light zones by CD1 mice during sequential exposures to the light/dark chamber at 25,000 lx (same experiments as in Fig. 2). On test days, animals were tested 30 min after injection of vehicle (PBS, i.p.) or CGRP (0.1 mg/kg i.p.). **A** Resting time in dark zone during the same experiment represented in Fig. 2A. Two-way (factors: time x treatment) ANOVA, Sidak’s multiple comparison test *p < 0.05, **p < 0.01 and ***p < 0.001 compared to corresponding time point in vehicle group. **B** Resting time in light zone during the same experiment represented in Fig. 2A. Two-way (factors: time x treatment) ANOVA (treatment factor *p* = 0.2743 for first test and *p* = 0.8969 for second test). **C** Resting time in dark zone during the same experiment represented in Fig. 2C. Two-way (factors: time x treatment) ANOVA, Sidak’s multiple comparison test *p < 0.05, **p < 0.01 and ***p < 0.001 compared to corresponding time point in vehicle group. **D** Resting time in light zone during the same experiment represented in Fig. 2C. Two-way (factors: time x treatment) ANOVA (ns)

As expected from our previous studies, mice from the first cohort showed an increased resting time in the dark after CGRP administration during both tests and independently of the phase of the circadian cycle (Fig. 5A), but no difference in resting time in the light (Fig. 5B). The second cohort however shows a less robust increased time resting in the dark, especially during the second test where significance is lost (*p* = 0.078) (Fig. 5C). Of note, the number of animals per group is lower than what we usually use for the light aversion assay, which could explain that significance is not quite reached. The time spent resting in the light was unchanged (Fig. 5D). The same observations can be made when data are plotted as averages and presented as scatter plots (Supplementary Figure 1).

### CGRP administration induces spontaneous pain independently of the time of the day

**Fig. 6:**
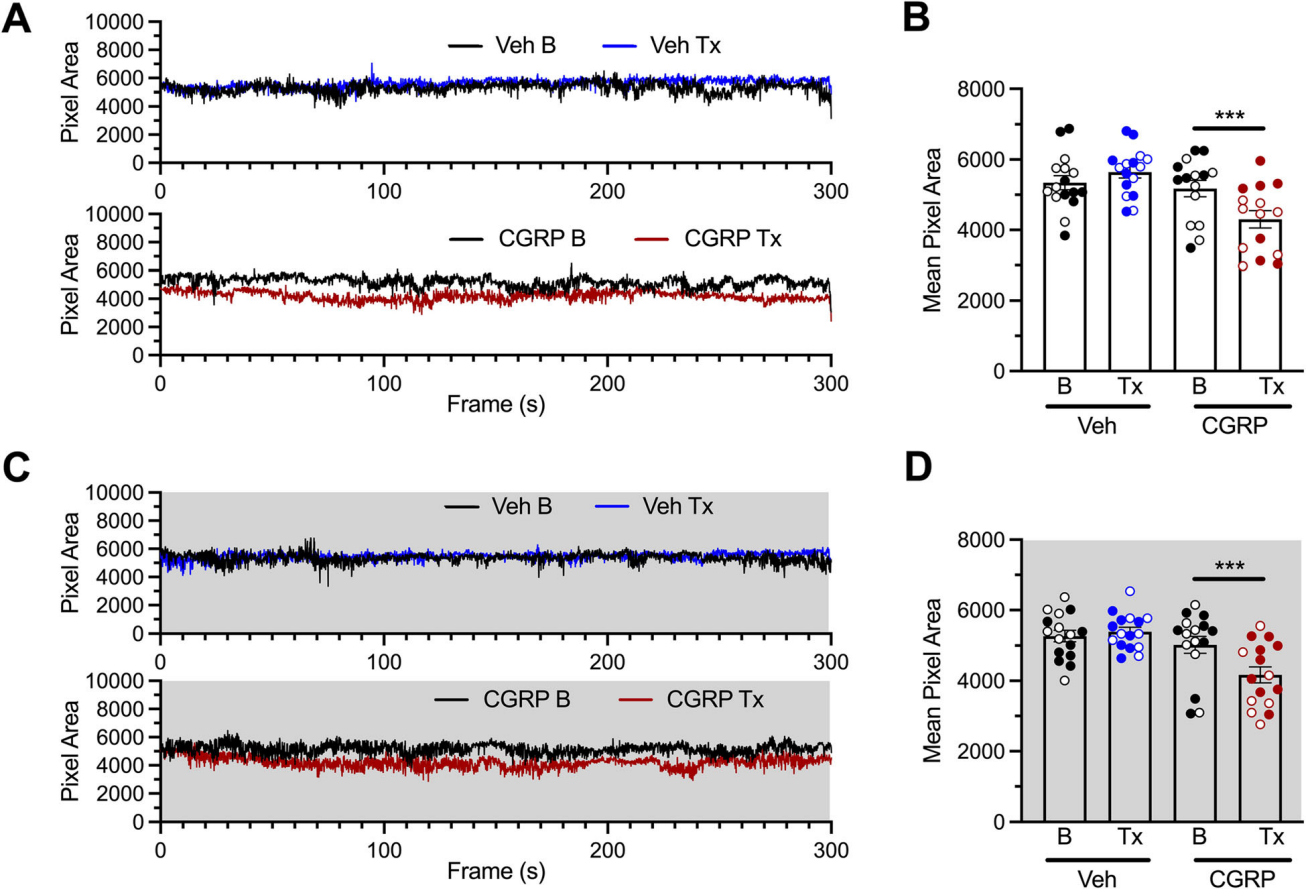
Peripheral CGRP decreases pixel area of the eye (increases the squint response) during both the active and inactive phases. **A** Mean pixel area over time during the light phase (10 AM) for each baseline (5 min recording, no injection) and treatment (5 min recording, 30 min post injection) for all mice injected with either Veh (PBS, *n* = 16, top) or CGRP (0.1 mg/kg, *n* = 15, bottom). **B** Mean overall pixel area from panel A ± SEM for all mice during baseline (B) and treatment (Tx) conditions. Empty symbols represent females, and full symbols represent males. There were no statistical differences between males and females. Two-way (factors: time x treatment) ANOVA, Sidak’s multiple comparison test ***p < 0.001 compared to corresponding baseline. **C** Mean pixel area over time during the night phase (10 AM) for each baseline (5 min recording, no injection) and treatment (5 min recording, 30 min post injection) for all mice injected with either Veh (PBS, *n* = 16, top) or CGRP (0.1 mg/kg, *n* = 15, bottom). **D** Mean overall pixel area from panel C ± SEM for all mice during baseline (B) and treatment (Tx) conditions. Empty symbols represent females, and full symbols represent males. There were no statistical differences between males and females. Two-way (factors: time x treatment) ANOVA, Sidak’s multiple comparison test ***p < 0.001 compared to corresponding baseline

The effect of the circadian cycle on spontaneous pain was investigated measuring squint, which is the principal component of the mouse grimace response [24]. All mice were tested both during the day (10 AM, light phase) and during the night (8 PM, dark phase). Half of the mice were tested first during the light phase and then during the dark phase, and the other half was reversed. Data were pooled by time of testing. Compared to their baseline, mice given vehicle during the light phase had the same pixel area indicating a lack of eye closure (Fig. 6A, top). Mice injected with CGRP during the light phase had decreased pixel area, indicating a squint response (Fig. 6A, bottom). The data for individual mice are shown in Fig. 6B. Similarly, when mice were injected at night, CGRP but not vehicle induced a significant decrease in the pixel area, indicating a squint response in the night phase (Fig. 6C and D). No sex differences were observed in this assay.

## Discussion

In this paper, we show that induction of migraine-like light aversion and squint response in mice by CGRP is not dependent on the circadian cycle. Consistent with a previous study [19], peripheral administration of CGRP in CD1 mice (which likely corresponds to an acute model of migraine) induced spontaneous pain, as well as light aversive behavior, accompanied by increased time spent resting in the dark, but not in the light. It is unclear at this point whether the time in the light and resting behaviors are linked or if increased resting is solely due to spontaneous pain. In fact, when facial signs of discomfort are assessed after peripheral administration of CGRP, the resulting grimace and squint that indicate spontaneous pain have been shown to be independent of light [24]. Considering that the light aversion assay relies on light, it is reassuring that testing in either the day or night is not a confounder of the behavioral output. As a control, the squint assay, which does not require any motility, is also independent of the phase of the circadian cycle. This assay has been designed to measure the eye opening (or squint) of mice, which is a translatable phenotype indicative of spontaneous pain [23, 24].

We also show that in two different assays (home cage movement and voluntary wheel running), activity is maximal during the first hours of the assay, with a similar burst of activity if tested during the active or inactive phase. This observation likely explains why all assays performed for the present study show that the CGRP-mediated effects are similar during the day and during the night, and therefore during the active and inactive phases of the circadian cycle. This indicates that the effects (or lack of thereof) of the cycle on motility are not sufficient to mask the migraine-like phenotypes induced by CGRP. It is noteworthy that mice were only tested up to three times each, with a recovery time in between. Therefore, it is likely that in this study, testing mice during their inactive phase only induced a modest sleep deprivation, if any, which in turn had no repercussion on the migraine-like symptoms. It would be interesting to repeat such experiments after inducing more robust sleep disturbances.

Multiple studies have reported the effect of circadian rhythms and light cycle on mice activity. Jhuang and colleagues noted a significant decrease in walking and hanging activities during the day time compared to night time in four different strains of mice [27]. Another study documented sheltering time, floor movement, and wheel activity (velocity and distance) over multiple days during both light and dark phases of the cycle, and similarly, reported that mice displayed a significantly greater activity at night across all parameters [28]. The 23-h ambulatory activity data in the present study corroborates those studies. The results obtained with the 2-h wheel assay were therefore surprising since there was no difference in the number of wheel revolutions when assessed during the day or during the night in vehicle administered animals. However, upon further investigation, this is not the first report a burst of activity during the first hour or two of an assay. In fact, de Visser and colleagues excluded Day 1 of their reporting because of “very high activity levels in the first hours after introduction to the cage” [28]. Similarly, in rats, a burst of running was observed in the hour after being placed into the testing cage [29]. In both studies though, the assay was always initiated at the same time of the circadian cycle, and no direct comparison of starting times could be done. In the present study, assays were started at different times of the cycle, yet yielded identical results during the first hours. Those results can likely be attributed to the initial burst of activity that would mask effects of the circadian cycle. In addition, handling mice during the day to place them in the wheel is likely to “wake them up”, explaining the similarity of the results during the day and night for short-term assays.

Depression in voluntary wheel activity has previously been used to characterize a migraine-like state in animal models. Dural administration of the TRPA1 agonist allyl isothiocyanate induced a dose-dependent reduction in wheel running in rats, which could be reversed by immediate administration of sumatriptan [30], and by pretreatment with Δ^9^-tetrahydrocannabinol in females [25]. The same team showed that depression in wheel activity could also be induced by repeated administration of morphine, mimicking medication overuse headache in rats [26]. In a 2016 study, Christensen et al., showed that infusion of nitroglycerin in rats failed to induce a decrease in time in motion and distance travelled using an in-cage wheel for 30 min [31]. The present data show that peripheral administration of CGRP decreases wheel running, which further supports the reliability of voluntary wheel activity as an indicator of pain in rodents. Future experiments are needed to determine whether traditional migraine treatments can attenuate this phenotype, and if lower doses of CGRP could reveal sex differences in this assay.

## Conclusions

We have previously shown that peripheral administration of CGRP in different strains of mice induced migraine-like symptoms such as light aversion [19], periorbital and plantar tactile sensitivity [32], and facial signs of discomfort [24]. A decrease in wheel activity is an additional clinically relevant phenotype observed in this model, which is reminiscent of the reduction in normal physical activity observed in migraine patients [33]. Those phenotypes are independent of the phase of the circadian cycle.

## Supplementary Information

Additional file 1: **Supplementary Figure 1.** Peripheral CGRP increases the time resting in the dark during both the active and inactive phases. In all panels, grey areas represent the active phase of the animals when lights of the facility were turned off (night). Data represent the average of resting time already presented in Fig. 5. **(A)** Resting time in dark zone. Unpaired t-test *p* = 0.936 for baseline, *p* < 0.0001 for Test 1, and *p* < 0.001 for Test 2. **(B)** Resting time in light zone. Unpaired t-test *p* = 0.925 for baseline, *p* = 0.528 for Test 1, and *p* = 0.839 for Test 2. **(C)** Resting time in dark zone. Unpaired t-test *p* = 0.816 for baseline, *p* = 0.015 for Test 1, and *p* = 0.077 for Test 2. **(D)** Resting time in light zone. Unpaired t-test *p* = 0.663 for baseline, *p* = 0.915 for Test 1, and *p* = 0.746 for Test 2.

## Data Availability

The datasets used and/or analyzed during the current study are available from the corresponding author on reasonable request.

## Abbreviations

CGRP: Calcitonin gene-related peptide
PBS: Phosphate buffer saline,
SEM: Standard error of the mean
