# Correction to: CGRP induces migraine-like symptoms in mice during both the active and inactive phases

**DOI:** 10.1186/s10194-021-01332-5

**Published:** 2021-10-05

**Authors:** Anne-Sophie Wattiez, Olivia J. Gaul, Adisa Kuburas, Erik Zorrilla, Jayme S. Waite, Bianca N. Mason, William C. Castonguay, Mengya Wang, Bennett R. Robertson, Andrew F. Russo

**Affiliations:** 1 Department of Molecular Physiology and Biophysics, University of Iowa, 51 Newton Rd, Iowa City, IA 52242 USA; 2 Center for the Prevention and Treatment of Visual Loss, Veterans Administration Health Center, Iowa City, IA 52246 USA; 3 Neuroscience Program, University of Iowa, Iowa City, IA 52242 USA; 4 Present Address: Brain and Behavior Sciences, Center for Advanced Pain Studies, University of Texas at Dallas, 800 West Campbell Rd, Richardson, TX 75080 USA; 5 Department of Pharmacology, University of Iowa, Iowa City, IA 52242 USA; 6 Department of Neurology, University of Iowa, Iowa City, IA 52242 USA

**Correction to: J Headache Pain 22, 62 (2021)**

**https://doi.org/10.1186/s10194-021-01277-9**

Following the publication of the original article [1], we were notified of an error in the spelling of the fourth author’s name.

- Originally published name: Erik Zorilla
- Corrected name: Erik Zorrilla

The original article has been corrected.
