# Supplementary figures and images for: CGRP induces migraine-like symptoms in mice during both the active and inactive phases
Source: J Headache Pain. 2021 Jun 30;22(1):62. doi: 10.1186/s10194-021-01277-9 (PMC8243868; doi:10.1186/s10194-021-01277-9)

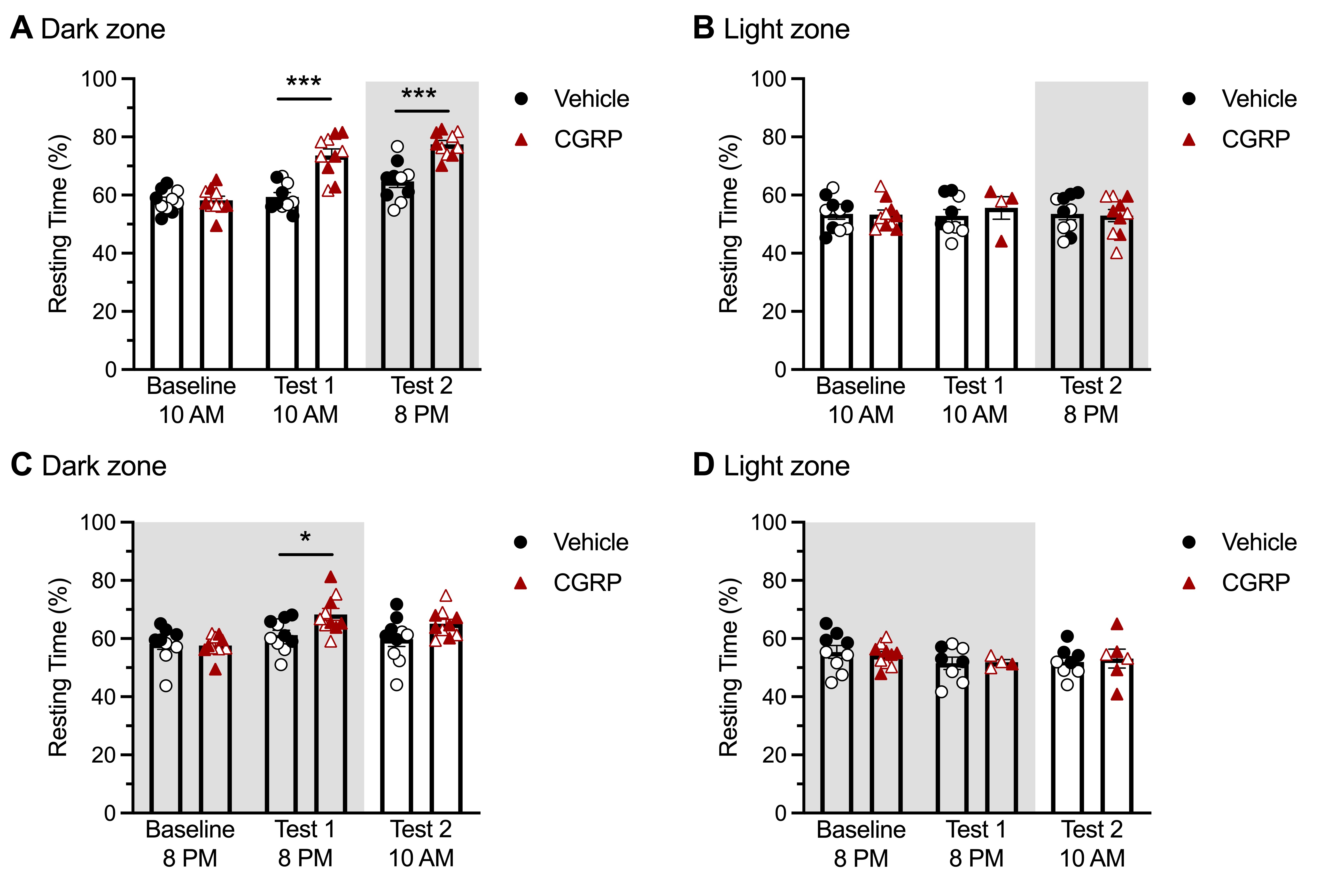

Supplement: Supplementary file 1 — Supplementary Figure 1. Peripheral CGRP increases the time resting in the dark during both the active and inactive phases. In all panels, grey areas represent the active phase of the animals when lights of the facility were turned off (night). Data represent the average of resting time already presented in Fig. 5. (A) Resting time in dark zone. Unpaired t-test p = 0.936 for baseline, p < 0.0001 for Test 1, and p < 0.001 for Test 2. (B) Resting time in light zone. Unpaired t-test p = 0.925 for baseline, p = 0.528 for Test 1, and p = 0.839 for Test 2. (C) Resting time in dark zone. Unpaired t-test p = 0.816 for baseline, p = 0.015 for Test 1, and p = 0.077 for Test 2. (D) Resting time in light zone. Unpaired t-test p = 0.663 for baseline, p = 0.915 for Test 1, and p = 0.746 for Test 2. [file 10194_2021_1277_MOESM1_ESM.jpg]
